# Polyaphron Formulations Stabilised with Different Water-Soluble Polymers for Ocular Drug Delivery

**DOI:** 10.3390/pharmaceutics14050926

**Published:** 2022-04-24

**Authors:** Roman V. Moiseev, Fraser Steele, Vitaliy V. Khutoryanskiy

**Affiliations:** 1 Reading School of Pharmacy, University of Reading, Whiteknights, Reading RG6 6DX, UK; r.moiseev@pgr.reading.ac.uk; 2 MC2 Therapeutics, 1A Guildford Business Park Rd., Guildford GU2 8XG, UK; fst@mc2therapeutics.com

**Keywords:** ocular drug delivery, cornea, polyaphrons, mucoadhesion, ocular irritation, water-soluble polymers

## Abstract

As drug delivery to the eye has evolved over the last decades, researchers have explored more effective treatments for ocular diseases. Despite this, delivering drugs to the cornea remains one of the most problematic issues in ophthalmology due to the poor permeability of the cornea and tear clearance mechanisms. In this study, four different types of polyaphron formulations are prepared with 10% poloxamer 188 (P188), 10% poly(2-ethyl-2-oxazoline), 1% polyquaternium 10, and 3% sodium carboxymethylcellulose solutions mixed with 1% Brij® L4 in a caprylic/capric triglycerides solution. Their physicochemical characteristics, rheological properties, and stability are assessed. Additionally, a polyaphron with 3% polyquaternium 10 was prepared for the assessment of ex vivo corneal retention along with four other polyaphrons. The best retention on the ex vivo cornea was displayed by the 3% polyquaternium 10-based formulation. The 10% poloxamer 188 along with 1% polyquaternium 10-based polyaphrons appeared to be the most stable among the four prepared formulations. A toxicological evaluation of these formulations was performed using a slug mucosal irritation test and bovine corneal opacity and permeability assay, with all four polyaphrons proving good biocompatibility with ocular tissues. The developed drug delivery systems demonstrated an excellent potential for ocular drug delivery.

## 1. Introduction

The statistics from the World Health Organisation indicate that over 2.2 billion people have vision impairment [1]. At the same time, diseases of the anterior eye segment are among the leading causes of corneal opacification. The delivery of drugs to the eye remains a problematic issue in ophthalmology, due to the poor permeability of cornea and nasolacrimal drainage [2]. Typical drug losses following a topical administration to the eye are very high, leading to only 2–3% of the intraocular absorption [3].

The topical administration of solutions and suspensions is the most common approach, which comprises over 70% of the commercially available ocular formulations [4]. Macro- and microemulsions are also used in ocular drug delivery and may offer advantages over aqueous solutions. These formulations comprise aqueous and oil phases with surfactants and co-surfactants [5,6,7]. In general, the non-ionic surfactants of a polymeric nature (e.g., poloxamers, polysorbates, polyethylene glycols, Brij^®^, and tyloxapol) are preferred over ionic surfactants, due to their lower toxicity. Amphoteric surfactants, such as lecithin, are also commonly used because of their low toxicity [8,9,10]. Additionally, the electrical double layer formed with ionic surfactants provides additional stabilisation for the emulsions or microemulsions. Therefore, the formulation stability is more affected by the ionic strength when ionic surfactants are used, rather than in the case of non-ionic surfactants being used [11]. On the other hand, the positively-charged surfactants and polymers may induce binding to the negatively charged epithelial cells of the cornea, potentially prolonging the precorneal retention time and, as a result, facilitating drug penetration. For instance, it was shown by Klang et al. that the spreading coefficient for the negatively charged emulsions on the corneas was four times lower than that of the positively charged emulsions [12].

The lipid phase of emulsions is commonly represented by long-chain triglycerides (LCTs), medium-chain triglycerides (MCTs), isopropyl myristate (IPM [13]), fatty acids (e.g., oleic acid), and oily sucrose esters [14]. MCTs are triglycerides with about 8–12 carbon atoms per hydrocarbon chain. A key advantage of MCTs over LCTs is their hydrophilicity, which is due to their higher density (0.94–0.95 g/cm^3^) being close to the density of water. As a result of their less lipophilic properties, MCTs are able to dissolve more drugs at higher concentrations than LCTs. Furthermore, the low viscosity of MCTs increases the concentration of the lipid phase within the emulsion. Typical examples of LCTs are vegetable oils (e.g., soybean oil and castor oil), whereas the most common MCT is Miglyol^®^ 812 (caprylic/capric triglycerides, CCT) [15,16,17]. Hence, a lipid phase of emulsions and microemulsions offers greater solubility for hydrophobic drugs, in comparison to aqueous formulations in ophthalmology. Additionally, emulsions and microemulsions may provide controlled drug-release kinetics and increased bioavailability. For example, Naveh et al. reported prolonged ocular hypotension in rabbits using a submicron emulsion with pilocarpine [18].

**Figure 1:**
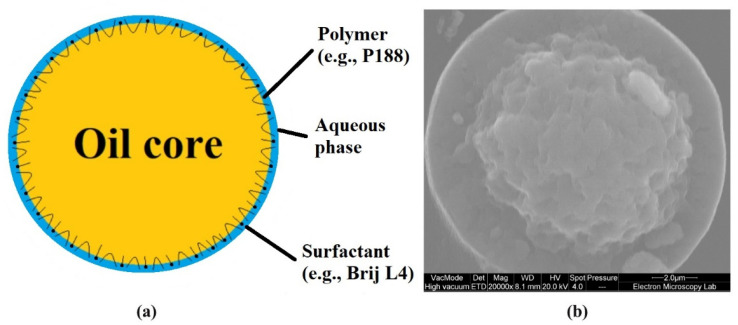
A schematic representation of a single polyaphron droplet containing an oil core (CCT) and thin water layer (**a**), and its cryo-SEM image (**b**).

Aphrons (also referred to as bi-liquid foams, foam-like emulsions, high internal phase emulsions (HIPEs), high internal phase ratio emulsions (HIPREs), or gel emulsions) are complex colloidal dispersion systems with droplets of oil/multiphase fluid or gas bubbles forming a core (dispersed phase) encapsulated in a thin layer of water that is stabilised by a water-soluble surfactant or polymer (continuous aqueous phase), which, in turn, may be dispersed in an additional aqueous phase. These colloidal systems were first described by Sebba in 1972, who also coined the term ‘aphron’ [19,20,21]. This word is derived from the Greek word ‘foam’ (αφρός-foam), due to the resemblance to a gas foam structure. These bi-liquid foams with an oil core (primarily MCTs) as a dispersed phase and continuous aqueous phase stabilised with a non-ionic/ionic surfactant or a polymer are designated as gel polyaphron dispersions (polyaphrons) [22,23]. Figure 1 shows a schematic representation of a single polyaphron droplet and its cryo-scanning electron microscopy (cryo-SEM) image.

By adding a continuous aqueous phase without any surfactant or polymer, the polyaphrons can be further diluted, producing so-called colloidal liquid aphrons [21,24,25,26]. The polyaphrons are often referred to as foam-like emulsions; nevertheless, these formulations can contain from ~70% to as much as ~90% of the dispersed oil phase and as less as 0.5% (reaching a maximum of 3%) of surfactant/polymer from the total weight of the formulation [22]. Therefore, polyaphrons have a much lower surfactant-to-oil ratio in comparison with conventional emulsions allowing for the reduction of the toxic effects on biological tissues [27].

This study aims to prepare four polyaphron formulations based on different water-soluble polymers, including 10% poloxamer 188 (P188), 10% poly(2-ethyl-2-oxazoline) (POZ), 1% polyquaternium 10 (PQ10), and 3% carboxymethylcellulose sodium salt (CMC) solutions mixed with Miglyol^®^ 812 containing Brij^®^ L4. These formulations are evaluated using the measurements of the droplet size, zeta potential, and pH values. Additionally, these polyaphrons are studied using cryo-scanning electron microscopy, and rheological and storage stability experiments. A toxicological assessment of four polyaphrons is conducted using a bovine corneal opacity and permeability test and a slug mucosal irritation test. Additionally, the polyaphron formulation with 3% PQ10 was prepared to be studied along with the above-mentioned four polyaphrons for its retention on bovine corneal tissues ex vivo, using the fluorescence-based flow through method. To the best of our knowledge, this is the first study reporting the comparison of four different water-soluble polymers used in the preparation of polyaphrons for ocular drug delivery.

## 2. Materials and Methods

### 2.1. Materials

Poloxamer 188 solution (P5556, 10% P188 solution); poly(2-ethyl-2-oxazoline) (372,846, POZ, Mw~50,000, PDI 3-4); polyquaternium 10 (525,944, PQ10); carboxymethylcellulose sodium salt (C5678, low viscosity, CMC); Brij^®^ L4 (235,989, Laureth-4); fluorescein sodium salt (F6377, NaFl); sodium bicarbonate (S6014); fluorescein isothiocyanate-dextran (FD4, FITC-dextran; Mw~3000–5000); and benzalkonium chloride (B6295, BAC) were purchased from Sigma-Aldrich Co. (Merck Life Science UK Limited; Gillingham, UK). Miglyol^®^ 812 (batch 130,802, caprylic/capric triglycerides, CCT) was obtained from Cremer Oleo GmbH & Co. (Hamburg, Germany), sodium chloride (S/3160/60), sodium hydroxide (S/4920/53), calcium chloride dihydrate (BP510-250), and phosphate-buffered saline tablets (12821680, PBS) were purchased from Fisher Scientific UK Ltd. (Loughborough, UK).

### 2.2. The Preparation of the Polyaphrons

Polyaphrons were prepared according to the protocol developed by MC2 Therapeutics Ltd., (Leatherhead, UK) [23]. This included a drop-wise addition with a Pasteur pipette of the oil phase (discontinuous) to the aqueous phase (continuous), which was first placed in the 250 mL laboratory beaker (with an internal diameter = 6.5 cm). Thus, 30 g of each product was formulated by stirring with a four-bladed impeller (diameter = 6.0 cm) set at 250 rpm (IKA Labortechnik RW20.n S2, IKA^®^ England Ltd., Oxford, UK). The initial speed of the drop-wise addition was around 1 drop every 7 seconds, but was increased once 10% of the discontinuous phase had been added. Highly viscous creamy white polyaphron formulations containing tightly packed individual aphrons were formed, as a result of this mixing. The exact pharmaceutical compositions are presented in Table 1. The concentrations of polymers in solutions were selected after optimisation, depending on the visual appearance of the aqueous solutions. Thus, the high solubility of both P188 and POZ in water allowed for the preparation of 10% solutions for preparing the polyaphrons. At the same time, the solubility of CMC and PQ10 was significantly lower, compared to P188 and POZ. Hence, the solutions with several concentrations were prepared for CMC (3% and 4%) and PQ10 (1%, 3%, and 5%). Since the viscosity of 4% CMC was very high, the less viscous 3% concentration was selected to prepare the consistency of the polyaphrons, comparable with that of the formulations based on P188 and POZ. The same selection process was performed to identify 1% as the most optimal concentration for the PQ10 solution. Nonetheless, 3% PQ10 solution was also used to prepare polyaphrons to be used as a positive control in mucoadhesion studies.

### 2.3. The Physicochemical Characterisation

#### 2.3.1. Cryo-SEM

Cryo-scanning electron microscopy was performed for four various samples (polyaphrons based on 10% P188 or 10% POZ or 1% PQ10 or 3% CMC solutions mixed with 1% Brij^®^ L4 in CCT solution) using an FEI Quanta 600 FEG SEM microscope (Field Electron and Ion Company, FEI, Hillsboro, OR, USA) and 2.4× T Microscope Server. The images were taken at a 2942× magnification. The sample preparation included a nitrogen slush plunge and vitrifying at −210 °C. The samples were platinum sputter-coated before imaging in the cryo-mode. The size measurements were conducted using ImageJ software (Version 1.50i, 2016, National Institutes of Health, Bethesda, MD, USA).

#### 2.3.2. The pH

The pH was measured for four samples of polyaphrons without dilution using a Mettler Toledo FiveEasy F20 pH meter (Mettler Toledo, Greifensee, Switzerland). These measurements were conducted in triplicate for each formulation.

#### 2.3.3. The Zeta Potential and Size Measurements

The zeta potential measurements were carried out for four polyaphron samples using Zetasizer Nano-ZS in disposable folded capillary cells DTS1070 (Malvern Instruments, Malvern Panalytical Ltd., Malvern, UK). Initially, each sample was diluted 10-fold with ultrapure water (Triple Red Ultra Pure Water System, Avidity Science, Long Crendon, UK) by adding 500 µL of a sample to 4500 µL of ultrapure water. Each sample was then diluted 25-fold with ultrapure water by adding 200 µL of the sample to 4800 µL of ultrapure water (the total dilution was 250 fold). Finally, each sample was shaken for 15 s using BioCote Stuart Vortex Mixer (Barloworld Scientific Ltd., Stone, UK), prior to characterisation. and was analysed 3 times at 25 °C, and the mean ± standard deviation values were calculated. Polyaphrons were kept at 4 °C overnight prior to measurements.

The size measurements of the same samples were performed using the Mastersizer 3000 (Malvern Instruments, Malvern Panalytical Ltd., Malvern, UK). The analysis was performed 5 times at 23 °C for each sample, and the mean ± standard deviation values were calculated (Table S1 in the Supplementary Materials). The polyaphrons were kept at 4 °C overnight, prior to these measurements.

#### 2.3.4. Rheology

During the rheological experiments, all the samples were prepared 48 h in advance and kept at 4 °C to reduce the potential influence of shear history on the rheological behaviour of the tested polyaphrons. The required amount of each polyaphron formulation was placed on the plate (25 °C) immediately before testing. The rheological assessments of four polyaphron formulations were performed using Anton Paar Modular Compact Rheometer MCR 102 (Anton Paar GmbH, Graz, Austria) with the parallel plate PP50 (49.965 mm in diameter, 1000 µm gap) and RheoCompass™ software (V1.21.652, Anton Paar, Graz, Austria). The rheological measurements of the viscosity change with the increase in the shear rate (*γ*) and shear stress were used to analyse the stability of the polyaphron samples under mechanical stress. All the rheological tests were conducted in triplicate for each sample.

#### 2.3.5. Stability

The polyaphron stability studies included a series of photographs of capped disposable cuvettes with polyaphron formulations obtained at different time points (from the day of preparation to the 35th day) using the camera of an iPhone XS at 2× magnification. The set of four polyaphron samples was prepared and placed into plastic cuvettes, which were stored vertically in a refrigerator at 4 °C. At the same time, another set was stored in an incubator at 37 °C.

### 2.4. The In Vitro Retention Test of the Polyaphrons on a Bovine Cornea

#### 2.4.1. The Preparation of the Simulated Tear Fluid (STF)

The simulated tear fluid (STF) was prepared according to the protocol described by Srividya et al. [28], using 0.670 g of sodium chloride, 0.200 g of sodium bicarbonate, and 0.008 g of calcium chloride dihydrate dissolved in 100 mL of deionised water at room temperature. The pH of the simulated tear fluid solution was adjusted to 7.40, as the pH of natural tear fluid is close to neutral [29,30]. The simulated tear fluid was kept at 37 °C during the retention experiments.

#### 2.4.2. The Mucoadhesion Test

The mucoadhesive properties of the five polyaphron samples (10% P188, 10% POZ, 1% PQ10, 3% CMC, and 3% PQ10) spiked with 0.5 mg/mL of fluorescein sodium salt, in comparison to the negative control (0.5 mg/mL of a FITC-dextran solution in deionised water), were assessed using ex vivo bovine cornea tissues, following a slightly modified protocol previously developed within our research group [31]. After the animals were slaughtered, the intact bovine eyeballs were provided by P.C. Turner Abattoirs (Farnborough, UK), packed, and transported in insulated plastic bags. Upon arrival, these eyes were visually assessed in terms of any tissue damage or corneal opacification. The corneas were carefully excised with a scalpel within 3 h of delivery, avoiding contact with their outer epithelial surface. Then, these dissected corneas were rinsed with 1 mL of freshly prepared simulated tear fluid and mounted on glass slides with the outer epithelial layer facing upward. Prior to the retention experiments in the incubator set at 34.5 °C [32], these slides with corneas were kept at 4 °C. Then, the tested material (20 μL of FITC-dextran solution or 20 mg of polyaphron formulations) was applied on the corneal surface and was subsequently irrigated with STF using a 60 mL syringe and a pump (Harvard Apparatus model 981074, Holliston, MA, USA) at a flow rate of 200 μL/min within 30 min. This flow rate was selected to exceed the normal tear production in human eyes, which is considered to be ~1–2 μL/min [29]. Fluorescence images of the corneas were acquired using a Leica MZ10F stereo-microscope (Leica Microsystems, Wetzlar, Germany) with the GFP filter-fitted Leica DFC3000G digital camera at a 0.8× magnification, and a 30 ms exposure time (gain 2.5×) at the intensity of 4. The acquired microscopy images were then analysed using ImageJ software (Version 1.50i, 2016) with the mean fluorescence values measured (after each irrigation with 1 mL of STF) with the subsequent calculation of the fluorescence intensity (%) for each time point, where the initial point was set as 100% (Table S2 in the Supplementary Materials). Prior to each wash-off experiment, the image of the tissue (cornea without test substance) was obtained to measure the blank tissue’s pixel intensity for the data normalisation. A histogram of the distribution of these fluorescence intensity values at different wash-time points (0 to 30 min with increments of 5 min) was represented as a function of a time with the calculated area under the curve (AUC) using OriginPro software (Version 2021; OriginLab Corporation, Northampton, MA, USA). All retention tests were conducted in triplicates for each formulation.

### 2.5. Toxicity Assessment

#### 2.5.1. Slug Mucosal Irritation Test (SMIT)

Slug mucosal irritation tests (SMITs) were performed following the protocol previously developed by our research group [33]. *Arion lusitanicus* slugs were collected in the Reading area (Reading, UK). These slugs were kept in a separate room in plastic containers at room temperature and were fed with cabbage, cucumber, lettuce, and carrots. The body linings of each slug were carefully examined, and only those without macroscopic injuries and with clear tubercles and a foot surface were used for the experiments. Prior to the experiments, slugs weighing ~6–24 g were selected and kept individually in 1.5 L glass beakers. These beakers were lined with paper-towel sheets soaked with 20 mL of PBS solution (pH = 7.40) and covered with a cling film perforated with the syringe needle allowing air ventilation. The slugs were kept in these beakers without food for 48 h at room temperature before being used in the experiments. Subsequently, each slug was individually weighed, followed by its placement into a 90 mm plastic Petri dish lined with Whatman™ filter paper (Maidstone, UK) soaked in positive or negative controls (2 mL of 1% BAC in PBS solution and 2 mL of PBS solution, respectively) or 2 g of 4 polyaphron formulations. As soon as the 60-minute contact period ended, the slugs were removed from the Petri dishes, rinsed with 10 mL of PBS solution, followed by gently wiping with a paper towel, and reweighed. The percentage of mucus production (MP%) was calculated using the following equation based on a slug body weight loss:

(1) $$\mathrm{MP}\%=\frac{\left(m_{b}-m_{a}\right)}{m_{b}}\times 100\%,$$

where m_b_ and m_a_ are the slug weights before and after exposure to the tested chemicals, respectively. The results of these experiments were presented as the mean ± standard deviation for each sample (*n* = 9).

#### 2.5.2. Bovine Corneal Opacity and Permeability (BCOP) Test

The cornea is normally resistant to the permeation of sodium fluorescein, due to the tight junctions and lipophilic nature of the epithelium [34]. When applied to the eye, some compounds may cause damage to the cornea by denaturing proteins or causing epithelial disruption. In turn, the corneal epithelium becomes more permeable, allowing sodium fluorescein to penetrate it. The irritation potential of various compounds is determined by the amount of sodium fluorescein that permeates the cornea. The damage to the epithelium also often results in corneal opacity, which indicates ocular toxicity. The cornea becomes opaque when damaged or scarred, which alters its colour. Therefore, the BCOP assay proposed by Gautheron et al. can be used for the evaluation of ocular toxicity of various chemicals [35]. In this work, the modified BCOP test was performed following a protocol previously described by Abdelkader et al. [34]. The assessment of the toxicological properties of four different polyaphron formulations was conducted in comparison with PBS solution (pH = 7.40) as the first negative control, 1M sodium hydroxide solution as the positive control (causing a substantial chemical burn), and 0.02% benzalkonium chloride (BAC) solution (the second negative control). BAC at 0.02% is commonly used in ~70% of commercial ophthalmic formulations as a preservative and penetration enhancer [36]. The freshly dissected eyeballs with the corneas facing upwards were wrapped with cling film and placed into plastic cups within 250 mL beakers. Prior to each experiment, the microscope image of the blank tissue (eyeball without test substance) was obtained to measure the pixel intensity of the blank tissue to take the tissue intrinsic fluorescence into account. For each eye, several drops of PBS solution were applied, and the beakers were covered with cling film and incubated in a water bath at 34 ± 1 °C for 10 min. On the top of the cornea, a silicone ring (8 mm diameter) was placed, and 0.1 mL/0.1 g of various test compounds (1M NaOH, PBS, 0.02% BAC, and 4 polyaphron formulations) were applied within the silicone ring. After 30 s, 10 mL of PBS solution was poured over to rinse off the test substance. Afterwards, the beaker was left in a water bath for another 10 min at 34 ± 1 °C. In the next step, it was removed from the bath, and the eyes were photographed using an iPhone XS camera at 2× magnification. A special stand was used to ensure that all the images were obtained from the same distance and angle. A 0.1 mL of 2% *w*/*v* sodium fluorescein solution was then added to the ring and left for 1 minute. Then, the NaFl solution was rinsed off with 10 mL of PBS solution after the ring removal. Then, the fluorescence images of the eyeballs were acquired using a Leica MZ10F stereo-microscope (Leica Microsystems, Wetzlar, Germany) with a GFP filter-fitted Leica DFC3000G digital camera at a 0.8× magnification, 130 ms exposure time (gain 1×) at the intensity of 3. The set of iPhone images was analysed using ImageJ software (Version 1.50i, 2016) with a subsequent 8 bit conversion for each image to measure the mean grey area (a.u.). All the fluorescence images were analysed using ImageJ software by calculating the mean fluorescence values with blank tissue luminescence figures subtracted (Table S3 in the Supplementary Materials). These data represent the relative fluorescent intensity (a.u.) of corneas pre-treated with various test substances, resulting in different permeability levels. The BCOP tests were conducted in triplicates for each formulation.

### 2.6. The Statistical Analysis

All the experiments were performed with several replicates (at least 3) and the mean values ± standard deviations were calculated and evaluated for significance using two-tailed Student’s *t*-test and one-way analysis of variance (ANOVA), followed by Bonferroni’s post hoc test using GraphPad Prism software (version 8.0.2; GraphPad Software Inc., San Diego, CA, USA), where *p* < 0.05 was set as the statistical significance criterion.

## 3. Results and Discussion

Four different water-soluble polymers were evaluated in the present study for their ability to stabilise polyaphron formulations. P188 is a non-ionic amphiphilic tri-block copolymer consisting of the middle block of polypropylenglycol and two side blocks of polyethyleneglycol. This polymer has been traditionally used in the preparation of polyaphrons [22,23]. Another non-ionic water-soluble polymer used in this study was POZ. This polymer is less amphiphilic compared to P188, but, recently, it received substantial attention from researchers for different pharmaceutical formulations, due to its unique physicochemical properties [37,38]. PQ10 is a quaternary ammonium salt of hydroxyethyl cellulose, which has a cationic nature. It is commonly used in hair conditioning products as an anti-static agent, film former, and fixative, as well as in ophthalmic formulations [39,40]. CMC is an anionic derivative of cellulose that is commonly used in pharmaceutical formulations as a viscosity-enhancing agent [41,42]. The structures of these polymers are shown in Figure S1 in the Supplementary Materials. Following some optimisation experiments, four polyaphron formulations were prepared and their physicochemical properties were studied.

### 3.1. The Physicochemical Characterisation

The average pH of all polyaphron formulations was determined to establish their compatibility with the ocular environment, taking into consideration that the pH levels of normal tears are 7.14–7.82. The pH of the tear fluid can also vary between 5.2 and 9.3, depending on the age and pathological conditions [29]. The results of the pH measurements are summarised in Table 2. The pH of polyaphron based on 10% P188 was 5.91 ± 0.26, while the pH values for POZ-based samples reached 6.37 ± 0.15. No statistically significant difference between the pH values of these two polyaphrons were observed. PQ10-based samples showed an average pH value of 6.02 ± 0.21, which was also not statistically different to the two polyaphrons stabilised with non-ionic polymers. The pH of the CMC-based polyaphron was 6.73 ± 0.09, which was significantly higher than pH values of P188- (*p* < 0.01) and PQ10-based (*p* < 0.05) polyaphron formulations. In general, topical ophthalmic formulations can be tolerated by the eye at pH levels between 3.5 and 9. Nonetheless, it is recommended to formulate ophthalmic preparations as close to the physiological pH of tear fluid as possible, for reducing the potential discomfort and increased lacrimation [43].

The average zeta potential (ζ) values for 250-fold diluted polyaphron formulations are presented in Table 2. These data are consistent with the nature of polymers used in these formulations. The P188- and POZ-based samples had a slightly negative values of zeta potential; perhaps this negative charge could result from traces of capric acid present in its triglycerides. Non-ionic polymers, in this case, reduce this charge. The polyaphron prepared with a cationic PQ10 had a positive value zeta potential, and the zeta potential of the CMC-based sample was negative due to its anionic nature. According to the literature data, it is suggested that the emulsions with zeta potential values ranging from −11 to −20 mV are close to the agglomeration threshold [44], while emulsions with zeta potentials greater than ±25 mV or more display good stability. However, steric factors due to the presence of polymers on surfaces could also play a significant role in the stabilisation of colloids.

The results of average size measurements of four polyaphron formulations using laser diffraction and cryo-SEM are presented in Table 2 and Table S1 in the Supplementary Materials. These data demonstrate that the size for the polyaphron droplets is around 5–8 µm, which is in a good agreement with the cryo-SEM images and the literature data [45]. No statistically significant difference is observed between the size of polyaphrons measured using laser diffraction and cryo-SEM (*p* > 0.05).

**Figure 2:**
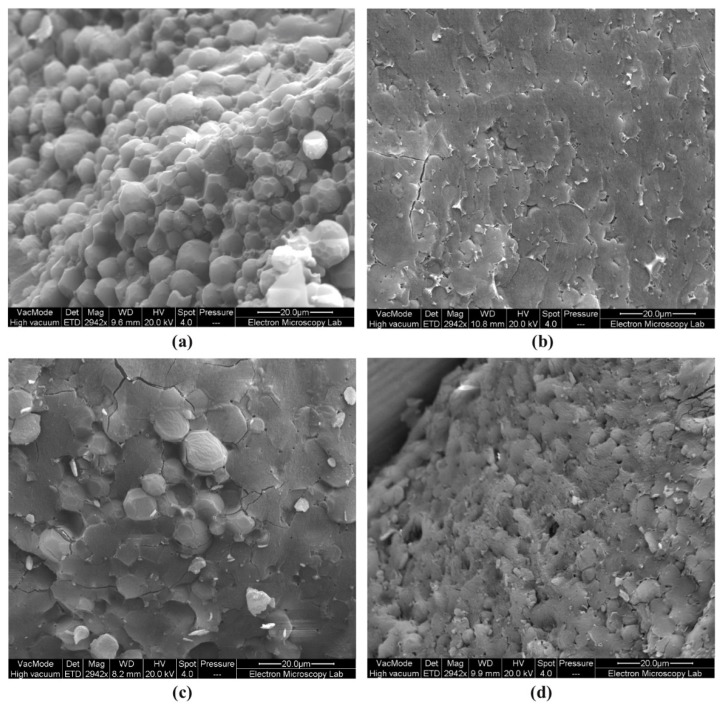
The exemplar cryo-SEM images of the polyaphron formulations with 10% P188 (**a**), 10% POZ (**b**), 1% PQ10 (**c**), and 3% CMC (**d**). Note that polyaphron droplets are not visible in the image (**b**), but can be clearly identified in an enlarged image (Figure S2 in the Supplementary Materials).

The cryo-SEM reveals a tightly packed gel polyaphron dispersion of a polyhedral shape for the samples based on 10% P188 (Figure 2a), and polyhedral structures for those with 1% PQ10 (Figure 2b). The distortion of the spherical shape can be explained with the oil volume fraction (φ_oil_) exceeding the values of 0.74, which is considered to be a maximum for the sphere-shaped solid, non-deformable, monodisperse solid structures [46]. However, the presence of a few cracks on the surface of the PQ10-based sample might demonstrate its lower stability compared to P188-based polyaphrons. At the same time, CMC-based samples (Figure 2d) also show polyhedron-shaped structures, but the stability of these polyaphrons seems to be lower than those with 1% PQ10. Finally, just a few distorted spheres can be found in the microphotographs of the POZ-based formulation (Figure 2c). These last formulations tend to be even more unstable, compared to the CMC-based samples. The size measurements conducted using the analysis of cryo-SEM images are also included in Table 2. Despite the general lack of cryo-SEM characterisation of polyaphrons in the literature, the published cryo-TEM images are consistent with the obtained images [45].

**Figure 3:**
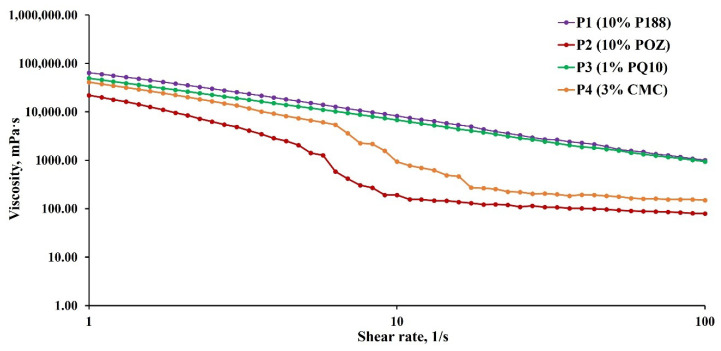
The rheological profiles of the four different polyaphron samples.

The stability of the pharmaceutical formulations is vitally important for their storage and transportation. The stability of four different polyaphron formulations was initially studied using rheological experiments (Figure 3). The rheological properties are generally affected by a variety of interacting factors, including the dispersed and continuous phases, the phase volume ratio, and the particle size distribution [44]. According to the rheological behaviour of the polyaphrons, they are classified as non-Newtonian fluids. This behaviour over a range of shear rates can be described with subsequent fitting using the power-law model (Ostwald–de Waele relationship) [47]:

(2) $$\eta =K\gamma^{\cdot n-1},$$

where *η* is the viscosity (mPa·s), *K* is the flow consistency index (mPa·s), *γ* is the applied shear rate (1/s), and *n* is the power law constant. The fluid is characterised as pseudoplastic when *n* < 1, Newtonian for *n* = 1, and dilatant for *n* > 1.

As the shear rate increases, a gradual decrease in the viscosity values can be observed for all four polyaphrons. Hence, polyaphrons display shear-dependent viscosity behaviour. The fitting with the power-law model is applicable to the polyaphrons with 10% P188 (*R*^2^ = 0.9478) and 1% PQ10 (*R*^2^ = 0.9841). Both of these formulations display shear-thinning behaviour with *n* = 0.3608 and 0.2643, respectively. These shear-thinning profiles are in a good agreement with the literature data [48]. Interestingly, the samples with 10% POZ and 3% CMC can be fitted using this model, only before a significant viscosity drop occurs for both of them at shear rates >4.79 s^−1^ for polyaphrons with 10% POZ and >6.31 s^−1^ for formulations with 3% CMC. This deviation from the power law is possibly related to the phase separation and lack of stability in these samples [47].

**Figure 4:**
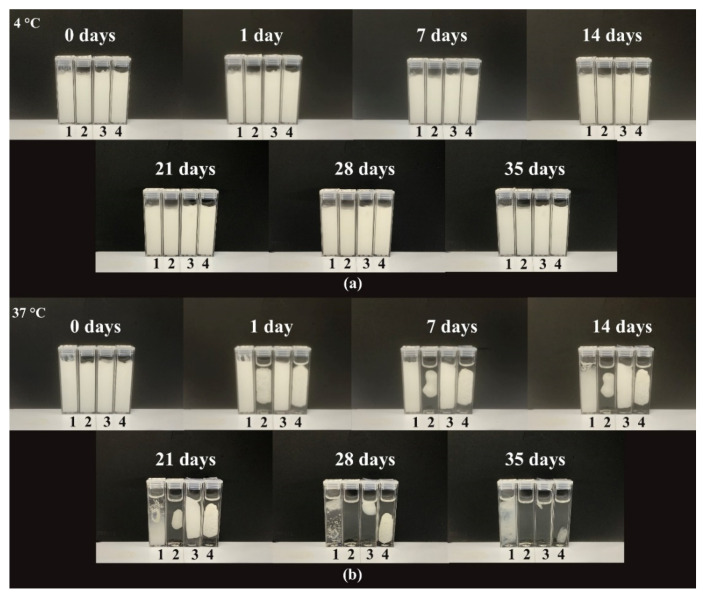
The stability studies of the polyaphrons with 10% P188 (1), 10% POZ (2), 1% PQ10 (3), and 3% CMC (4) stored at 4 °C (**a**) and at 37 °C (**b**) for 35 days.

In addition to the rheological and zeta-potential measurements, the stability of the polyaphrons was assessed by an analysis of the sample appearance, using a series of photographs taken at different time points (Figure 4). The set of samples stored at 4 °C displayed good stability and did not show any visual signs of phase separation, even after 35 days of storage. However, different behaviour was observed for the polyaphrons kept at 37 °C. Thus, both the POZ- and CMC-based samples demonstrated a phase separation after the first 24 h at 37 °C. The formulation with 1% PQ10 appeared to be more stable, still showing minor signs of phase separation after 24 h from its preparation. In contrast to the three previously mentioned formulations, the P188-based polyaphrons displayed excellent stability for 7–8 days, and then started showing some first signs of instability and phase separation. These changes visually appear as a gelation process of the P188, which might not have previously occurred, due to a relatively low concentration of P188 (1% by weight of the total weight of the formulation) [49]. According to our stability results, it can be concluded that all four polyaphrons remain stable when no mechanical stress is applied or elevated temperature is used for their storage, with P188-based samples overperforming in comparison to the other formulations.

These storage stability data correlate well with the rheological properties of the polyaphrons. The good stability observed for the samples prepared using 10% P188 may be related to the amphiphilic nature of this polymer, with more hydrophobic poly(propylene glycol) blocks being dissolved in the oil phase at the lipid–water interface. The non-ionic POZ-based formulation showed one of the worst stability results, which is perhaps related to the lack of amphiphilicity as well as its relatively low molecular weight. At the same time, the formulations with 3% CMC demonstrated poor stability, such as the POZ-based samples. This is possibly because the CMC macromolecules are very hydrophilic and are not able to dissolve in the oil even partially. The PQ10 macromolecules are also very hydrophilic; however, the stability of polyaphrons on their basis is greater in comparison to the CMC-based samples. Given the fact that PQ10 macromolecules are positively charged, the observed stability might be explained by their electrostatic attraction to a negatively charged oil surface. Therefore, the stability of the four various polyaphron samples can be arranged in the following decreasing order: P188 > PQ10 > CMC > POZ. Although there is not much rheological data available for the polyaphrons with the total surfactant concentration up to 2% (by weight of total composition) and size of around 10 μm, it can be concluded that the selection of polymers probably has a key role in polyaphron formulation stability, which is enabled by the modification of the interfacial and continuous phase rheology.

### 3.2. The In Vitro Retention on the Bovine Cornea

**Figure 5:**
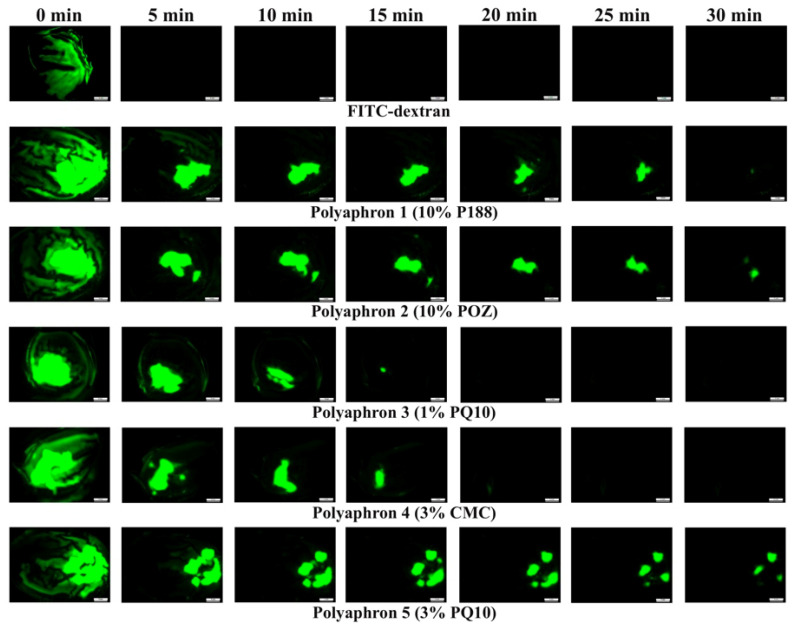
The exemplar images of the bovine corneas with applied FITC-dextran, and polyaphrons with 10% P188, 10% POZ, 1% PQ10, 3% CMC, and 3% PQ10. The brightness of the FITC-dextran exemplar images was increased with Procreate^®^ (Version 5.2.4; Savage Interactive Pty Ltd., Nipaluna, Australia). The numerical values are presented in Table S2 in the Supplementary Materials. Scale bars are 5 mm.

**Figure 6:**
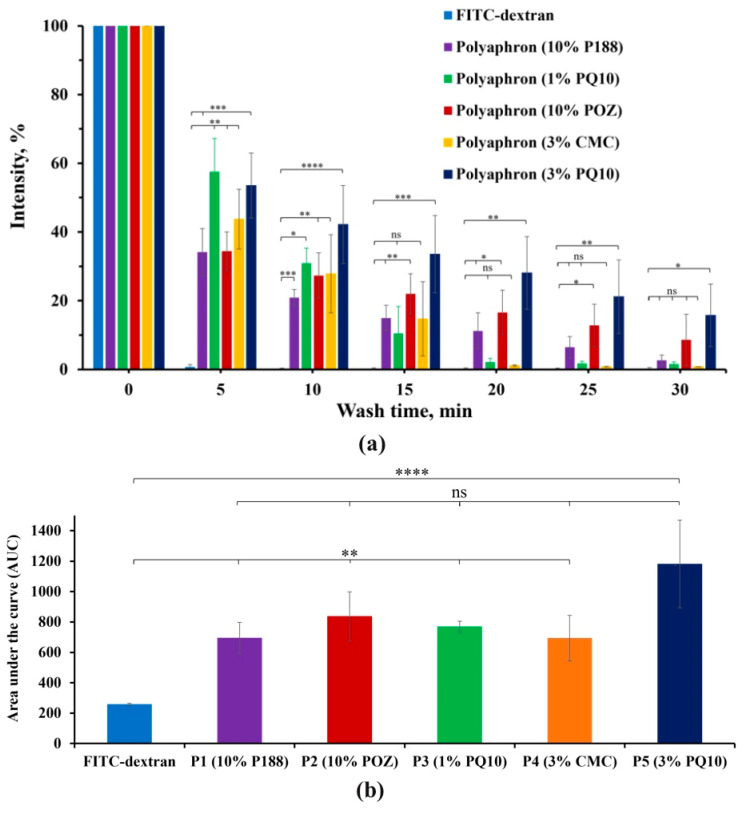
The assessment of the retention properties for the polyaphrons with 10% P188, 10% POZ, 1% PQ10, 3% CMC, and 3% PQ10, in comparison to FITC-dextran in deionised water using a wash-off test for 30 min (**a**). Area under the retention curve values represent the mucoadhesive properties of FITC-dextran and these polyaphrons (**b**). The percentage data set is expressed as the mean ± standard deviation (*n* = 3). The statistically significant differences are represented as: **** *p* < 0.0001; *** *p* < 0.001; ** *p* < 0.01; * *p* < 0.05; ns: no significance.

One of the first uses of water-soluble polymers to enhance the retention properties of drugs on ocular surfaces was proposed by Swan [50]. Since then, various strategies have been applied to improve corneal drug retention [51]. In this study, the retention properties of five various polyaphron samples in comparison to FITC-dextran dissolved in deionised water (negative control) were evaluated using ex vivo bovine corneas, and following a slightly modified wash-off test previously reported by our research group [31]. Exemplar fluorescence images are shown in Figure 5. These images were then converted into numerical values using image analysis (Figure 6a). Following the first 5 min of washing with STF, there was a significantly greater retention of all five polyaphron formulations, compared to the negative control, but no difference was observed between the polyaphron formulations. Similar levels of retention were observed after 10 min. Surprisingly, only 10% P188-, 10% POZ-, and 3% PQ10-based samples showed statistically significant differences, compared to the FITC-dextran solution after 15 min of washing off with *p* < 0.01 for the 10% P188- and 10% POZ-based polyaphrons, and *p* < 0.001 for the formulation with 3% PQ10. At the same time, similar retention behaviour for these three polyaphrons was also observed in the next 5 min, whereas, after 25 min, the polyaphrons with 10% POZ and 3% PQ10 were the only samples demonstrating a statistical difference, compared to the negative control with *p* < 0.05 and *p* < 0.01, respectively. At the final time point of 30 min, the 3% PQ10-based sample was the only formulation demonstrating a statistically significant difference (*p* < 0.05). It is likely that the 3% PQ10-based sample showed the best mucoadhesive properties due to the presence of a positively charged PQ10, which will electrostatically interact with negatively charged mucins [52]. However, the results for the sample with a lower concentration of PQ10 were not statistically significantly different after 15 min of the wash-off test, which might be explained by the low concentration of PQ10 within the formulation (0.1% by weight of the total weight). At the same time, CMC-based polyaphrons demonstrated relatively poor retention, such as the 1% PQ10-based polyaphron. However, according to the literature data, CMC is expected to display superior mucoadhesive properties, compared to the poloxamer-based formulations [51]. The concentration of CMC used in our formulation by weight of the total composition was 0.3%. Even though the CMC concentration reported to be used in ophthalmic preparations ranges from 0.2% to 2.5%, this might not be enough to present an enhanced mucoadhesive performance [43]. For instance, Garrett et al. reported that 0.5% CMC binds to human corneal epithelial cells [42]. Moreover, this effect is dose-dependent, as 1% CMC demonstrates greater retention properties [53]. At the same time, Paugh et al. reported that the ocular retention of the 3.5% low-viscosity CMC formulation on the ocular surface is up to ~10 min [54]. On the other hand, increasing the anionic polymer concentration in the formulation may cause more ocular irritation. At the same time, both the P188- and POZ-based formulations showed a prolonged corneal retention. Both of these compositions are based on non-ionic polymers and have similar viscosity values, as determined in the previous rheological studies. Remarkably, their viscosity values were much lower than those for the 1% PQ10-based sample. It is generally believed that a greater formulation viscosity facilitates better retention on the preocular surface.

The calculation of the area under the curve (AUC) for all the time points (0 to 30 min, with increments of 5 min) demonstrated a statistically significant difference between FITC-dextran and the 3% PQ10-based (*p* < 0.0001) sample, and 4 other polyaphrons (*p* < 0.01) (Figure 6b and Table S1 in Supplementary Materials). Interestingly, no statistical difference was observed between the AUCs of all the polyaphron formulations.

### 3.3. The Toxicity Assessment

Ocular tissues are very delicate and, when new drug delivery systems are developed, there is a strong need to establish the non-irritant nature of the formulations. Historically, the Draize rabbit eye test has been considered as the gold standard for evaluating acute ocular toxicity [55]. However, its use has ethical, scientific, and regulatory concerns [56]. Thus, the modern ethical characterisation of the formulations aims to minimise in vivo animal testing [57]. Furthermore, the recent toxicology development has enabled the evaluation of the ocular toxicity of various compounds, replacing the Draize test with a combination of various ex vivo and in vitro tests [58,59]. Several alternative methods, such as the bovine corneal opacity and permeability (BCOP) test, hen’s egg test on the chorioallantoic membrane, isolated rabbit eye test, isolated chicken eye test, and the slug mucosal irritation test (SMIT), have been proposed as alternatives to the Draize test [60,61,62,63]. In the present study, the biocompatibility of polyaphrons was analysed using the BCOP and SMIT methods.

#### 3.3.1. The Slug Mucosal Irritation Test

**Figure 7:**
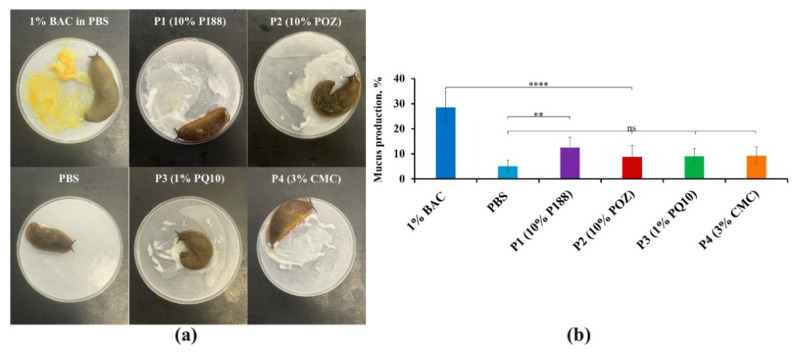
The exemplar images of the slugs exposed to 1% BAC in PBS, PBS solution, polyaphrons with 10% P188, 10% POZ, 1% PQ10, and 3% CMC (**a**), and the slug’s mucus production (**b**) after 60 min of contact with these formulations using the slug mucosal irritation test. The data set is expressed as the mean ± standard deviation (*n* = 9). The statistically significant differences are represented as: **** *p* < 0.0001; ** *p* < 0.01; ns: no significance.

The SMIT was developed by Adriaens et al. for the evaluation of the mucosal irritancy potential of various pharmaceutical compositions and excipients by measuring a slug’s mucus production (MP%) [64,65]. A slightly modified version of this test is routinely used by our research group [33,66]. Exemplar images of slugs after 60 min of exposure to 1% BAC in PBS (positive control), PBS solution (negative control), polyaphrons with 10% P188; 10% POZ; 1% PQ10; and 3% CMC, together with the mucus production values are presented in Figure 7. As expected, 1% BAC in PBS solution demonstrated a severe irritation potential, with MP% reaching 29 ± 6%. A significant amount of variability in the results of the experiment with the positive control is explained by the slugs’ increased activity and tendency to avoid contact with irritant chemicals. These results are in good agreement with the previous data published by Adriaens et al. and Kaldybekov et al. [64,66]. The PBS solution used as a negative control resulted in 5 ± 2% of mucus production, which is in agreement with the previously reported results by Adriaens et al. as well as Khutoryanskaya et al. [33,64]. At the same time, there was no statistically significant difference between the negative control and polyaphrons with 10% POZ (9 ± 5%), 1% PQ10 (9 ± 3%), and 3% CMC (9 ± 3%), indicating that these formulations do not cause any irritation effects. However, significantly higher values of mucus production were observed in the case of the P188-based sample (12 ± 4%), compared to the PBS solution (*p* < 0.01). This is possibly related to the presence of P188. Nonetheless, according to the literature data, P188 is classified as a minor ocular irritant [67]. Furthermore, it is FDA approved and extensively used in commercially available ophthalmic formulations (e.g., in Blephagel^®^ by Théa, Clermont-Ferrand, France and Cationorm^®^ eye drops by Santen, Osaka, Japan).

#### 3.3.2. The Bovine Corneal Opacity and Permeability (BCOP) Test

**Figure 8:**
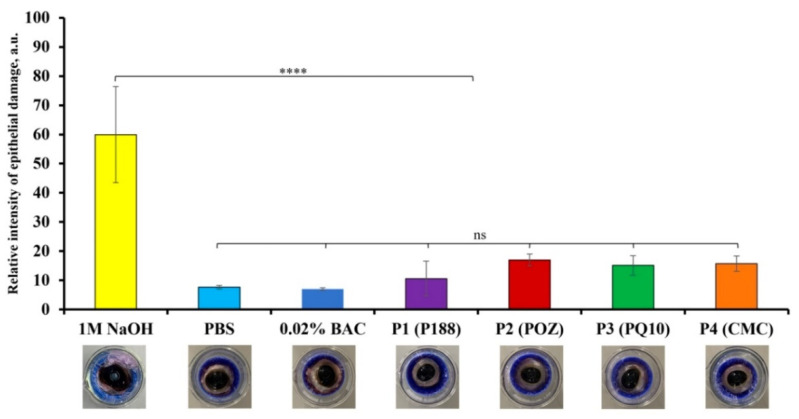
The epithelial damage demonstrated for 1M NaOH, PBS, 0.02% BAC solutions, polyaphrons with 10% P188, 10% POZ, 1% PQ10, and 3% CMC with exemplar images below each sample, respectively. The relative intensity of the epithelial damage values are expressed as the mean ± standard deviation (*n* = 3). The statistically significant differences are represented as: ****—*p* < 0.0001; ns: no significance.

The BCOP test was used to evaluate the toxicological properties of the four polyaphrons, in comparison to the PBS solution (pH = 7.40; negative control), 1M NaOH solution (positive control), and 0.02% BAC solution. Figure 8 and Figure S3 in the Supplementary Materials present the results of the opacity test. It is clear that 1M NaOH causes the highest corneal opacity (59.95 ± 16.46 a.u.), which is in agreement with the literature [68]. In contrast, the opacity value for the exposure of the bovine cornea to the PBS solution is 7.64 ± 0.55 a.u., and it is comparable to the result recorded for the 0.02% BAC solution (7.00 ± 0.47 a.u.). BAC, at this concentration, is commonly used in many ocular formulations [36]. Given the possible differences in the sensitivity between the cameras that were used, it can be concluded that the obtained results for the control solutions are generally comparable with the previously reported data [69]. According to our results (Table S3 in the Supplementary Materials), the opacity values observed after the exposure of the bovine corneas to the tested polyaphrons with 10% P188 (10.58 ± 5.98 a.u.), 10% POZ (16.96 ± 2.02 a.u.), 1% PQ10 (15.09 ± 3.35 a.u.), and 3% CMC (15.67 ± 2.61 a.u.) are significantly smaller, in comparison to the 1M NaOH (*p* < 0.0001), and show no difference when compared to both negative controls.

**Figure 9:**
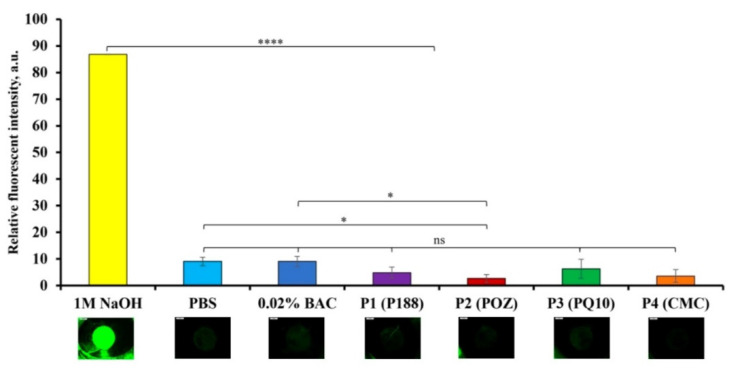
The relative fluorescent intensity values recorded for the bovine corneas exposed to NaFl following their exposure to 1M NaOH, PBS, 0.02% BAC solutions, polyaphrons with 10% P188, 10% POZ, 1% PQ10, and 3% CMC with exemplar images below each sample, respectively. The values are presented as the mean ± standard deviation (*n* = 3). The statistically significant differences are represented as: **** *p* < 0.0001; * *p* < 0.05; ns: no significance.

Figure 9 and Figure S4 in the Supplementary Materials summarise the results recorded for the permeability test. The fluorescence values (Table S3 in the Supplementary Materials) recorded for the bovine corneas, following their exposure to 1M NaOH, are the highest across all the tested substances (86.84 ± 0.10 a.u.). The fluorescent intensity values recorded following the exposure of the bovine corneas to the PBS and 0.02% BAC solutions are very similar (9.05 ± 1.59 a.u. and 9.06 ± 1.89 a.u., respectively). No statistically significant difference was observed between both negative controls, but there is a significant difference between the negative and positive controls (*p* < 0.0001). These permeability data are broadly comparable with the previously reported results [68,69]. Additionally, there was a substantial difference between 1M NaOH and the four different polyaphrons (*p* < 0.0001). No significant difference was observed between both negative controls and the P188- (4.85 ± 2.10 a.u.), PQ10- (6.29 ± 3.56 a.u.), and CMC-based (3.59 ± 2.36 a.u.) polyaphrons. Interestingly, the the POZ-based samples showed slightly lower intensity values than both negative controls with 2.66 ± 1.52 a.u. (*p* < 0.05). Considering that each eye is different, there may be some variability in the corneal thickness, which may impact the results.

BCOP and SMIT data collected on four polyaphrons indicated that each formulation does not cause any toxicological effects, demonstrating their suitability for application in ocular drug delivery.

## 4. Conclusions

In this study, four different polyaphron formulations stabilised with 10% poloxamer 188, 10% poly(2-ethyl-2-oxazoline), 1% polyquaternium 10, and 3% sodium carboxymethylcellulose solutions mixed with 1% Brij^®^ L4 were prepared. Their physicochemical characteristics, rheological, ex vivo corneal retention, and stability were assessed. The best retention ability was displayed by the 3% PQ10-based composition. The 10% P188- and 1% PQ10-based polyaphrons appeared to be the most stable among the prepared formulations. Additionally, a biocompatibility evaluation was performed using SMIT and BCOP assays, with all four polyaphrons proving low toxicity for ocular tissues. The obtained characteristics of the developed drug delivery systems with a total concentration of polymers <2% demonstrated their good potential for ocular drug delivery. This study indicated that the amphiphilicity of a water-soluble polymer is very important in the stabilisation of polyaphrons. However, when the positively charged polymer was used for the stabilisation of the polyaphrons, this allowed for the improvement of their retention on ocular surfaces. The present study demonstrated the possibility of preparing polyaphrons using water-soluble polymers of different natures, and the structural features and helped to identify some of the factors affecting their stability and mucoadhesive properties. Polyaphrons are an excellent platform technology for preparing various pharmaceutical formulations that could be used not only in ocular drug delivery, but in many other areas of transmucosal administration. The present study reports the evaluation of drug-free formulations. When these systems are used to formulate a particular active pharmaceutical ingredient, there is a possibility that drug molecules will have an additional influence on the physicochemical properties and stability of polyaphrons.

## Figures and Tables

**Table 1:** The calculated ratios for the ingredients in the polyaphrons with 10% P188, 10% POZ, 1% PQ10, 3% CMC, and 3% PQ10 from the total weight of the pharmaceutical composition.

Polyaphrons	Oil Phase (Discontinuous), %		Aqueous Phase (Continuous), %				
	CCT	Brij^®^ L4	Water	P188	POZ	PQ10	CMC
P1 (10% P188)	89.1	0.9	9	1	-	-	-
P2 (10% POZ)	89.1	0.9	9	-	1	-	-
P3 (1% PQ10)	89.1	0.9	9.9	-	-	0.1	-
P4 (3% CMC)	89.1	0.9	9.7	-	-	-	0.3
P5 (3% PQ10)	89.1	0.9	9.7	-	-	0.3	-

**Table 2:** The physicochemical characteristics of the polyaphrons with 10% P188 (P1), 10% POZ (P2), 1% PQ10 (P3), and 3% CMC (P4).

Polyaphrons	pH (*n* = 3)	Zeta Potential, mV (1:250 Diluted, *n* = 3)	Size, μm (Mastersizer, *n* = 5)	Size, μm (Cryo-SEM, *n* = 20)
P1 (10% P188)	5.91 ± 0.26	−17.8 ± 0.5	7.02 ± 0.03	8.15 ± 1.54
P2 (10% POZ)	6.37 ± 0.15	−11.3 ± 0.5	6.38 ± 0.19	6.74 ± 2.10
P3 (1% PQ10)	6.02 ± 0.21	29.8 ± 1.3	7.80 ± 0.01	8.21 ± 2.50
P4 (3% CMC)	6.73 ± 0.09	−25.6 ± 0.4	5.62 ± 0.01	5.49 ± 0.36

## Data Availability

All raw data are included in Supporting Information or from the authors.

## Supplementary Materials

The following supporting information can be downloaded at: https://www.mdpi.com/article/10.3390/pharmaceutics14050926/s1, Figure S1: Structures of poloxamer 188 (P188) (a), poly(2-ethyl-2-oxazoline) (POZ) (b), polyquaternium 10 (PQ10) (c), sodium carboxymethylcellulose (CMC) (d), Brij® L4 (Laureth-4) (e); Table S1: The size measurements of polyaphrons with 10% P188 (P1), 10% POZ (P2), 1% PQ10 (P3), and 3% CMC (P4) using Mastersizer 3000 (*n* = 5). DV 50 (Mass Median Diameter (MMD) or the median of the volume distribution) is the droplet size in microns at which 50% of the analysed sample is smaller and 50% is larger; Dv 10—the droplet size below which 10% of the sample lies; Dv 90—the droplet size below which 90% of the sample lies; Figure S2: Enlarged image of polyaphron formulation with 10 % POZ. Exemplar polyaphron droplets are highlighted with red circles (note that these circles do not show the actual diameter of these droplets); Figure S3: BCOP images used for opacity analysis following bovine corneas exposure to 1M NaOH, PBS, 0.02% BAC solutions and polyaphrons with 10% P188, 10% POZ, 1% PQ10, and 3% CMC; Figure S4: Fluorescence images of bovine corneas after administration of sodium fluorescein following tissue exposure to 1M NaOH, PBS, 0.02% BAC solutions, and polyaphrons with 10% P188, 10% POZ, 1% PQ10, and 3% CMC. Scale bars are 5 mm; Table S2: Average fluorescence (a.u.), intensity and AUC values for the retention test of four polyaphrons with 10% P188 (P1), 10% POZ (P2), 1% PQ10 (P3), and 3% CMC (P4) (*n* = 3); Table S3: Relative intensity of the epithelial damage (opacity) (a.u.) and relative fluorescence intensity of sodium fluorescein permeability (a.u.) values for the BCOP test of four polyaphrons with 10% P188 (P1), 10% POZ (P2), 1% PQ10 (P3), and 3% CMC (P4) (*n* = 3).
