# Supplementary material for: Polyaphron Formulations Stabilised with Different Water-Soluble Polymers for Ocular Drug Delivery
Source: Pharmaceutics. 2022 Apr 24;14(5):926. doi: 10.3390/pharmaceutics14050926 (PMC9146855; doi:10.3390/pharmaceutics14050926)
Supplement: Supplementary file 1 [file pharmaceutics-14-00926-s001.zip › pharmaceutics-1651981-supplementary.pdf]

pharmaceutics

MDPI

Supplementary information to

Article

Polyaphron Formulations Stabilised with Different Water-Soluble Polymers for Ocular Drug Delivery

Roman V. Moiseev[1], Fraser Steele[2] and Vitaliy V. Khutoryanskiy[1,*]

[1] Reading School of Pharmacy, University of Reading, Whiteknights, PO box 224, Reading RG66DX, United Kingdom; R.Moiseev@pgr.reading.ac.uk
[2] MC2therapeutics; Fst@mc2therapeutics.com
* Correspondence: v.khutoryanskiy@reading.ac.uk; Tel.: +44(0)118 378 6119.

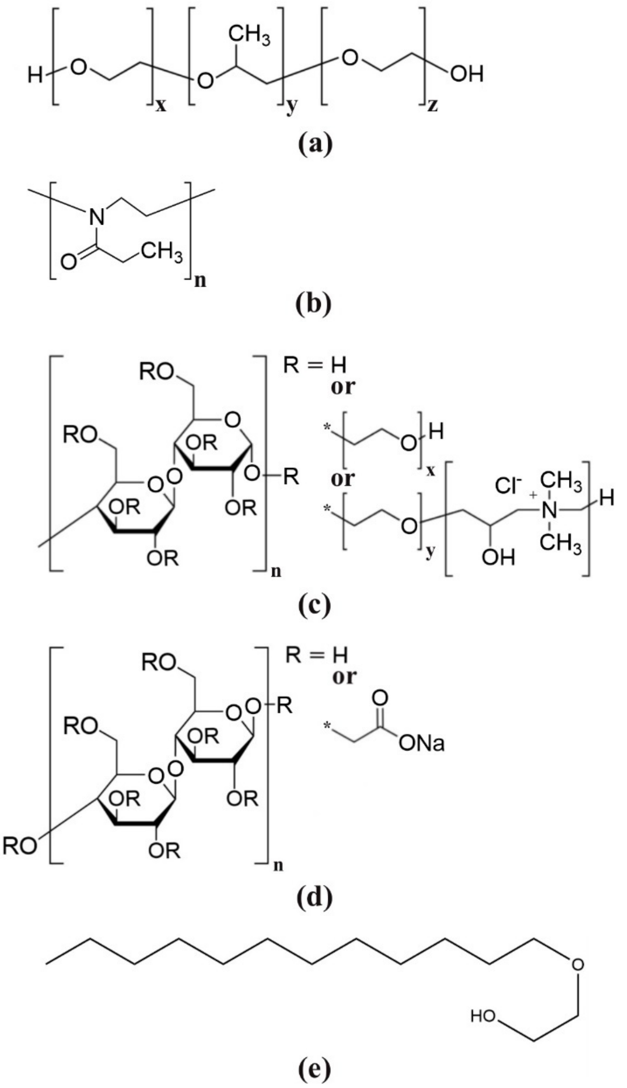

Figure S1. Structures of poloxamer 188 (P188) (a), poly(2-ethyl-2-oxazoline) (POZ) (b), polyquaternium 10 (PQ10) (c), sodium carboxymethylcellulose (CMC) (d), Brij® L4 (Laureth-4) (e).

Table S1. The size measurements of polyaphrons with 10% P188 (P1), 10% POZ (P2), 1% PQ10 (P3), and 3% CMC (P4) using Mastersizer 3000 (n = 5). DV 50 (Mass Median Diameter (MMD) or the median of the volume distribution) is the droplet size in microns at which 50% of the analysed sample is smaller and 50% is larger; Dv 10 – the droplet size below which 10% of the sample lies; Dv 90 – the droplet size below which 90% of the sample lies.

Polyaphrons	Dv 10, µm	Dv 50, µm	Dv 90, µm	Uniformity
P1 (10% P188)	1.74 ± 0.13	7.02 ± 0.03	11.80 ± 0.19	0.394
P2 (10% POZ)	1.56 ± 0.07	6.38 ± 0.19	11.50 ± 0.31	0.446
P3 (1% PQ10)	4.19 ± 0.01	7.80 ± 0.01	12.42 ± 0.04	0.338
P4 (3% CMC)	1.27 ± 0.01	5.62 ± 0.01	11.34 ± 0.09	0.542

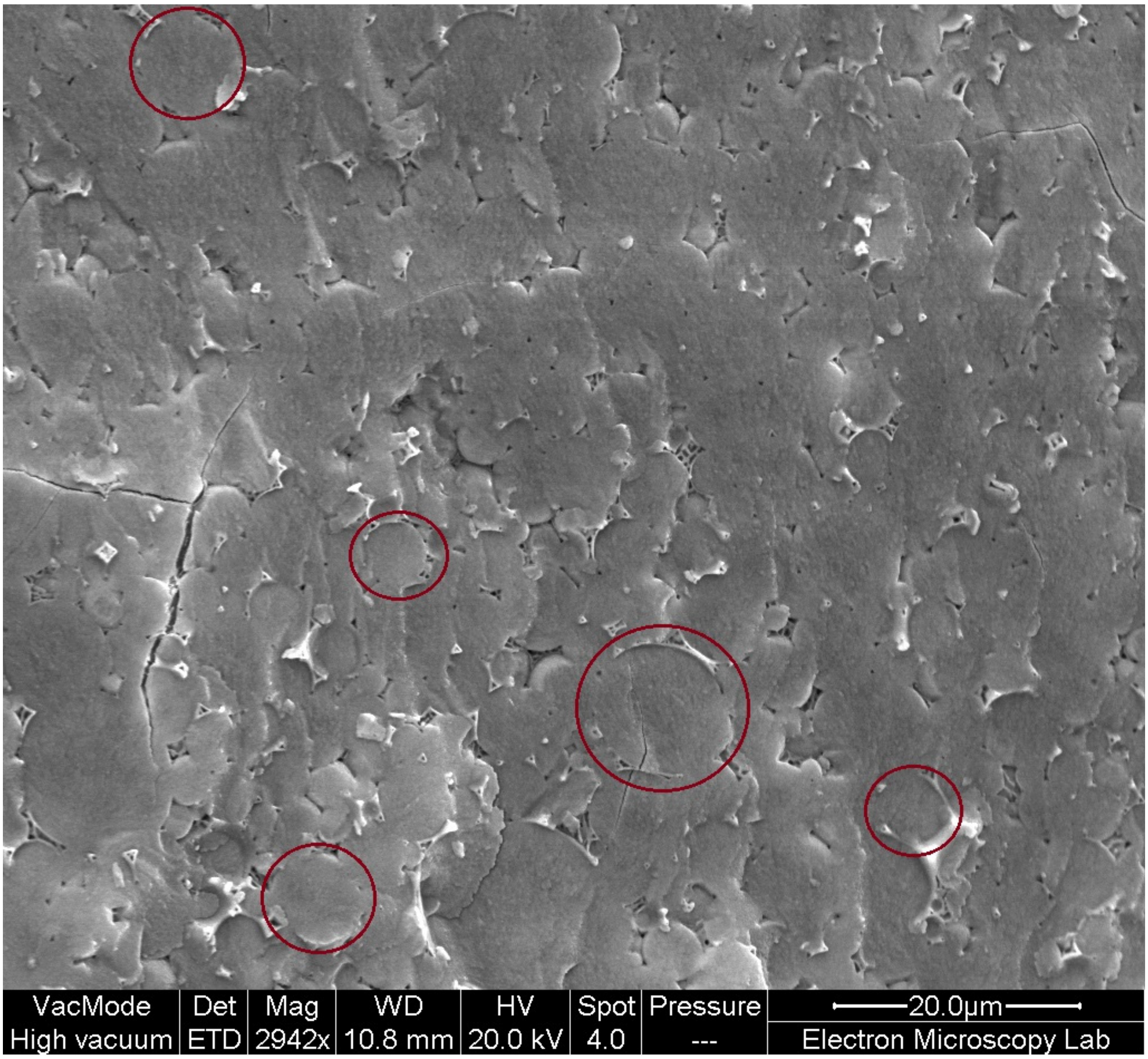

Figure S2. Enlarged image of polyaphron formulation with 10 % POZ. Exemplar polyaphron droplets are highlighted with red circles (note that these circles do not show the actual diameter of these droplets).

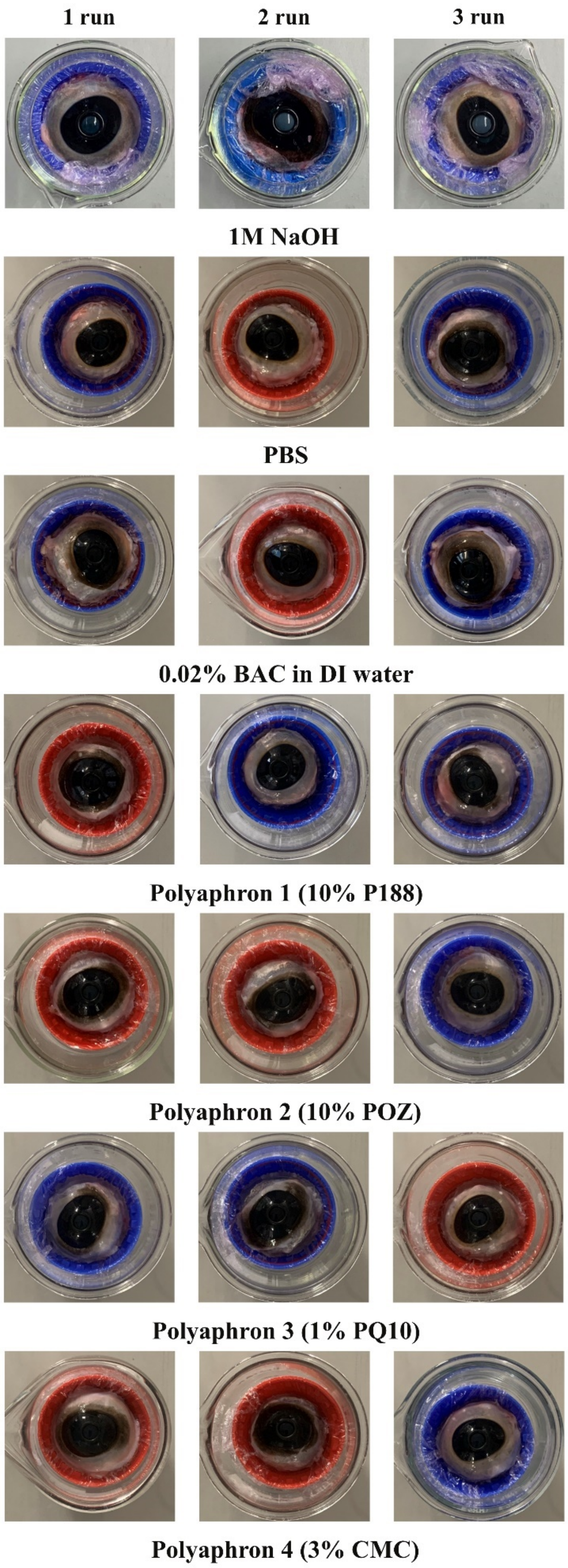

Figure S3. BCOP images used for opacity analysis following bovine corneas exposure to 1M NaOH, PBS, 0.02% BAC solutions and polyaphrons with 10% P188, 10% POZ, 1% PQ10, and 3% CMC.

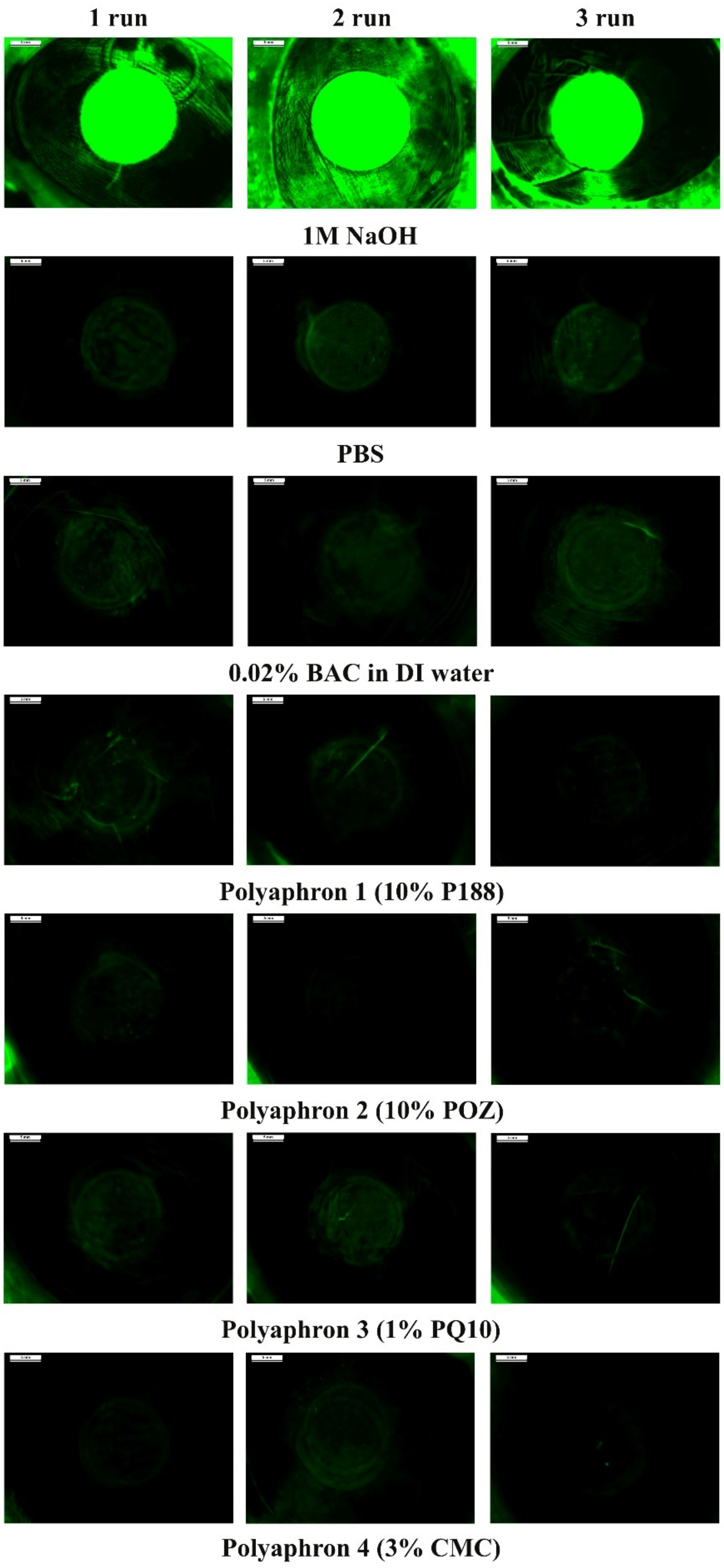

Figure S4. Fluorescence images of bovine corneas after administration of sodium fluorescein following tissue exposure to 1M NaOH, PBS, 0.02% BAC solutions, and polyaphrons with 10% P188, 10% POZ, 1% PQ10, and 3% CMC. Scale bars are 5 mm.

Table S2. Average fluorescence (a.u.), intensity and AUC values for the retention test of four polyaphrons with 10% P188 (P1), 10% POZ (P2), 1% PQ10 (P3), and 3% CMC (P4) (n = 3).

	Time, min	FITC-dextran	P1 (10% P188)	P2 (10% POZ)	P3 (1% PQ10)	P4 (3% CMC)
**Average fluorescence values, a.u.**	0	1.482 ± 0.388	35.078 ± 7.141	25.012 ± 1.365	15.961 ± 1.806	20.849 ± 5.447
	5	0.011 ± 0.011	12.266 ± 4.939	8.574 ± 1.266	9.108 ± 1.092	8.816 ± 1.069
	10	0.002 ± 0.003	7.432 ± 2.318	6.823 ± 1.665	4.963 ± 1.034	5.402 ± 0.753
	15	0.003 ± 0.004	5.324 ± 1.960	5.506 ± 1.582	1.671 ± 1.297	2.687 ± 1.231
	20	0.003 ± 0.004	4.084 ± 2.413	4.167 ± 1.747	0.329 ± 0.125	0.248 ± 0.095
	25	0.002 ± 0.004	2.321 ± 1.296	3.216 ± 1.643	0.272 ± 0.086	0.166 ± 0.049
	30	0.003 ± 0.006	1.005 ± 0.797	2.193 ± 1.958	0.249 ± 0.087	0.161 ± 0.034
**Intensity, %**	0	100 ± 0	100 ± 0	100 ± 0	100 ± 0	100 ± 0
	5	0.739 ± 0.667	34.125 ± 6.831	34.383 ± 5.627	57.566 ± 9.649	43.739 ± 8.686
	10	0.140 ± 0.220	20.908 ± 2.327	27.328 ± 6.582	30.948 ± 4.352	27.843 ± 11.376
	15	0.200 ± 0.226	14.970 ± 3.691	21.987 ± 5.883	10.505 ± 7.849	14.721 ± 10.828
	20	0.200 ± 0.226	11.208 ± 5.242	16.575 ± 6.458	2.129 ± 1.051	1.160 ± 0.194
	25	0.129 ± 0.217	6.437 ± 3.078	12.798 ± 6.181	1.733 ± 0.638	0.796 ± 0.075
	30	0.159 ± 0.413	2.656 ± 1.548	8.626 ± 7.455	1.578 ± 0.598	0.779 ± 0.051
**AUC**		257 ± 8	695 ± 104	837 ± 162	768 ± 37	693 ± 150

Table S3. Relative intensity of the epithelial damage (opacity) (a.u.) and relative fluorescence intensity of sodium fluorescein permeability (a.u.) values for the BCOP test of four polyaphrons with 10% P188 (P1), 10% POZ (P2), 1% PQ10 (P3), and 3% CMC (P4) (n = 3).

	**1M NaOH**	**PBS**	**0.02%BAC**	**P1 (10%P188)**	**P2 (10%POZ)**	**P3 (1%PQ10)**	**P4 (3%CMC)**
**Relative intensity of the epithelial damage (opacity), a.u.**	59.95 ± 16.46	7.64 ± 0.55	7.00 ± 0.47	10.58 ± 5.98	16.96 ± 2.02	15.09 ± 3.35	15.67 ± 2.61
**Relative fluorescence intensity of NaFl permeation (permeability), a.u.**	86.84 ± 0.10	9.05 ± 1.59	9.06 ± 1.89	4.85 ± 2.10	2.66 ± 1.52	6.29 ± 3.56	3.59 ± 2.36
